# Supplementary material for: Shear-Wave-Elastography in Neurofibromatosis Type I
Source: Diagnostics (Basel). 2022 Jan 31;12(2):360. doi: 10.3390/diagnostics12020360 (PMC8871512; doi:10.3390/diagnostics12020360)
Supplement: Supplementary file 1 [file diagnostics-12-00360-s001.zip › diagnostics-1522253-supplementary.pdf]

Parameter	Neurofibromatosis Type 1 (NF 1)	Neurofibromatosis Type 2 (NF 2)	Comparison NF 1 vs. NF 2
SWV of PNT/median nerve in lower arm (m/s)	2.8±0.8 (1.6-4.9)	3.8±1.7 (2.5-6.2)	U= 14.0, p=0.329
SWV of median nerve in extension (m/s)+	4.4±1.5 (2.0-7.4)	4.6±0.5 (4.3-5.3)	U=21.0, p=0.949
SWV of median nerve in neutral position (m/s) +	2.8±0.7 (1.9-4.0)	2.9±0.6 (2.2-3.7)	U=18.0, p=0.642
SWV of median nerve in flexion (m/s) +	2.7±0.6 (1.9-3.8)	2.3±0.4 (2.45-6.2)	U=14.0, p=0.343
Selected PNT length in lower arm (mm)	11.9±8.0 (1.4-27.8)	7.5±4.4 (3.5-13.7)	U=12.0, p=0.226
Selected PNT height in lower arm (mm)	3.1±1.9 (1.0-7.4)	3.7±1.3 (2.5-5.0)	U=15.0, p=0.396
Selected width PNT in lower arm (mm)	4.5±3.0 (1.4-12.2)	3.8±1.4 (3.0-5.9)	U=19.0, p=0.731

Supplemental Table S1. Results for SWE in patients with neurofibromatosis type 1 and 2.
Mean ± standard deviation, (range of all values).
+ different positions are demonstrated in Figure 2.
SWV: shear wave velocity
PNTs: peripheral nerve tumors
